# Addition of Intrathecal Fentanyl or Meperidine to Lidocaine and Epinephrine for Spinal Anesthesia in Elective Cesarean Delivery

**DOI:** 10.5812/aapm.14081

**Published:** 2014-02-07

**Authors:** Farnoush Farzi, Ali Mirmansouri, Kambiz Forghanparast, Abtin Heydarzadeh, Mehrsima Abdollahzadeh, Fatemeh Jahanyar Moghadam

**Affiliations:** 1 Deaprtment of Anesthesiology, Guilan University of Medical Sciences, Rasht, Iran; 2 Department of Microbiology, Guilan University of Medical Sciences, Rasht, Iran; 3 Department of Community Medicine, Guilan University of Medical Sciences, Rasht, Iran

**Keywords:** Anesthesia, Spinal, Meperidine, Fentanyl, Pain, Postoperative Cesarean Section

## Abstract

**Background:**

A common and useful approach to pain management is administration of neuraxial opioids.

**Objectives:**

Whether addition of fentanyl or meperidine to lidocaine and epinephrine for spinal anesthesia in elective cesarean delivery has any effects on duration of postoperative pain.

**Patients and Methods:**

This was a clinical trial, conducted on 195 pregnant women candidates for elective cesarean section. All patients were in ASA classes I, and II aged 17-45 years, and were randomly allocated to three groups named as meperidine (P), fentanyl (F), and placebo (S). In the three groups (P, F, and S), 25 mg meperidine, 25 µg fentanyl and 0.5 mL saline with lidocaine and epinephrine were injected into the subarachnoid space for spinal anesthesia, respectively. Perioperative complications and Apgar scores were recorded. Duration of analgesia was measured from the end of operation for 24 hours by using VAS. The first VAS≥4 was recorded as the end of the painless period. Characteristics of sensory and motor block were assessed. Statistical analysis was performed with SPSS software.

**Results:**

The mean duration of analgesia with meperidine, fentanyl or placebo were 9.46 ± 0.6, 6.27 ± 0.45, 2.06 ± 0.13 hours, respectively (P < 0.0001). There was significant difference between the group P and the other groups. Patients on meperidine had faster, longer and higher sensory block (P < 0.0001) and faster and longer motor block (P < 0.0001). Frequency of sedation in the group F was more than the others (P < 0.026). There was no difference in Apgar scores between the three groups (P < 0.45).

**Conclusions:**

Addition of meperidine or fentanyl to lidocaine and epinephrine solution increases the duration of postoperative analgesia in cesarean section. Meperidine is a recommended adjuvant according to longer duration of analgesia and lower complications.

## 1. Background

Cesarean section is one of the most commonly performed operations in women. Technique of anesthesia depends on the indication of cesarean section, emergency level, maternal hemodynamic status, and her preference (1). Postoperative pain results increased basic muscular tone in the site of operation, reduced muscular functioning, physical immobility, and delayed returning of the patient's performance (2). 

The use of general anesthesia for cesarean delivery has declined during the recent years, but it is still necessary for some situations and is performed with different combinations of drugs (3).

Regional techniques, especially spinal anesthesia, have long been known as safe and trustable methods to provide anesthesia for various surgical procedures (4). Regional techniques are increasingly recommended in obstetrics anesthesia due to lack of systemic drug effects on mother and fetus (5).

After discovering opioid receptors on spinal cord, postoperative pain management has encountered new aspects (6), and different combinations of local anesthetics, opioids, and other adjuvants were used for spinal block (7).

## 2. Objectives

This study was performed to compare the analgesic effects of intrathecal fentanyl and meperidine added to lidocaine and epinephrine to provide spinal anesthesia for cesarean section. We also compared the side effects of these drugs including changes in blood pressure, nausea, vomiting, sedation and respiratory depression in mothers during the first 24 hours after the cesarean section. Apgar scores of the newborns were recorded at the first and fifth minutes post-delivery.

## 3. Patients and Methods

This double-blinded, randomized, placebo-controlled study was conducted in Al-Zahra Teaching Hospital in Rasht, after approving by the Ethical Committee of Guilan University of Medical Sciences under the number 1901022504, and registering at the Iranian Registry of Clinical Trials under the number IRCT201201128677 N2. One hundred and ninety five pregnant women aged 17-45 with ASA classes 1 and 2 planned to undergo cesarean delivery were enrolled after providing complete verbal information about the study and the drugs, and obtaining a written consent. Pregnant women did not enter the study in any condition that spinal anesthesia was contraindicated like patient refusal, patient's inability to maintain stillness during the needle puncture, raised intracranial pressure, coagulopathy, skin or soft tissue infection at the proposed site of needle insertion, and severe hypovolemia. They also did not enter the study if they had a known allergy/ hypersensitivity to opioids or local anesthetics, pregnancy induced hypertension, chronic pruritus, chronic use of narcotics or substance abuse, multiple gestation and emergency cesarean section due to maternal and fetal causes like placenta previa or fetal distress. Exclusion criteria included simultaneously two or more operations, operation time longer than 1.5 hour, and blood loss more than 1500 milliliters. 

In preoperative period, patients were randomly assigned to three groups: group P = meperidine, group F = fentanyl, and group S = saline by picking a card from a bag. The letters on the cards determined the drug that would be administered to each patient. The patients and the persons, who evaluated postoperative pain, did not know which drug was administered to the patient. Before spinal anesthesia, an 18 gauge intravenous cannula was inserted for all patients and they received 10 ml/kg normal saline, and standard monitoring was applied including ECG, HR, NIBP, and pulse oximetry (SAADAT Digital Monitoring). Spinal anesthesia was administered in sitting position by an expert anesthesiologist (15 years of experience) using a 25-gauge Quincke needle through L3-L4 or L4-L5 intervertebral spaces. 

 Lidocaine 70 milligrams and epinephrine 0.1 milligrams were administered intrathecally plus 25 mg meperidine in group P, 25 µg fentanyl in group F, and 0.5 mL normal saline in group S. Total volume of administered drug was 2 milliliter in all patients. After the spinal injection, patients were immediately placed in supine position with left uterine displacement. Supplemental oxygen was administered via a simple face mask at a rate of 5-8 L/min. A level of sensory block to T6 by pinprick (bilateral mid axillary line using a short beveled 27-guage needle performed at 2,4,6,8,10 and 15 minutes following subarachnoid injection and then every fifteen minutes until regression to T10) was considered satisfactory for the operation. The onset time of complete motor block was assessed after assessing sensory block by a modified Bromage scale (0 = no paralysis, 1 = unable to raise extended leg, 2 = unable to flex knee, 3 = unable to flex ankle) (8) every 3 minutes for 15 minutes and then every 10 minutes until complete motor recovery. Perioperative complications including blood pressure alterations, nausea, vomiting, sedation, respiratory depression, pruritus and neonatal Apgar scores were recorded by another anesthesiologist with no role in administering spinal anesthesia. After administration of spinal anesthesia, blood pressure was assessed every 3 minutes for 20 minutes and then every 5 minutes until the end of operation. Any drop in blood pressure was treated with an intravenous bolus dose of 5 mg ephedrine, repeated as necessary, to maintain systolic blood pressure within 20% of the baseline values to the maximum dose of 30 mg. Phenylephrine 200 µg was administered if there was not any appropriate response to ephedrine. Bradycardia, if occurred during the operation, was treated with atropine 0.5 mg intravenously. After delivery, all patients received an infusion of 30 International Units of oxytocin intravenously during one hour. During the first 24 hours after the operation, patients' pain and possible complications like pruritus, sedation and respiratory depression were assessed every hour for the first 6 hours and then every 2 hours for the next 18 hours by an anesthesiologist. A visual analog scale (VAS) of 4 or higher was considered as pain and was treated with Diclofenac suppository (50 mg). This time was regarded as the end of analgesia period. Nausea or vomiting was treated with 0.1 mg/kg metoclopramide intravenously. If pruritus occurred, 25 mg promethazine was administered intravenously and if it was not effective, 0.08 mg naloxone was administered intravenously. If respiratory depression occurred (respiratory rate less than 9 per minute), 0.08 mg naloxone was administered intravenously, and if it was not effective, a dose of 0.04 mg naloxone was administered again.

The level of consciousness was determined by the Ramsay Sedation Scale. Score 4 or higher was regarded as sedation (9). 

### 3.1. The Ramsay Sedation Scale

1. Patient is anxious and agitated or restless or both.
2. Patient is cooperative, oriented and tranquil.
3. Patient responds to commands only.
4. Patient exhibits brisk response to light glabellar tap or loud auditory stimulus.
5. Patient exhibits a sluggish response to light glabellar tap or loud auditory stimulus.
6. Patient exhibits no response.

Finally, collected data was analyzed. Quantitative variables were analyzed by 

Kruskal–Wallis, log rank and Kaplan Mayer Tests, and qualitative variables were analyzed with Chi square test by using SPSS 16 software. A P value less than 0.05 was considered statistically significant. 

## 4. Results

One hundred ninety five pregnant women candidates for elective cesarean section under spinal anesthesia were randomly enrolled, and all of them completed the study. Patients' demographic characteristics were similar in all the three groups (Table 1). 

**Table 1.** Demographic Characteristics of Patients

Variable	Placebo (n = 65), No. (%)	Meperidine (n = 65), No. (%)	Fentanyl (n = 65), No. (%)	Total (n = 195), No. (%)	P value
**Age, y**					
< 30	31 (47.7)	41 (63.1)	44 (67.7)	116 (59.5)	0.052
≥ 31	34 (52.3)	24 (36.9)	21 (32.3)	79 (40.5)	0.052
Mean ± SD	32.16 ± 7.31	28.64 ± 6.14	27.73 ± 6.01	29.51 ± 6.76	0.0001
**Weight, kg, Mean ± SD**	85.1 ± 12.19	77.95 ± 9.56	81.69 ± 8.82	81.58 ± 10.85	0.0001

**Figure 1.**
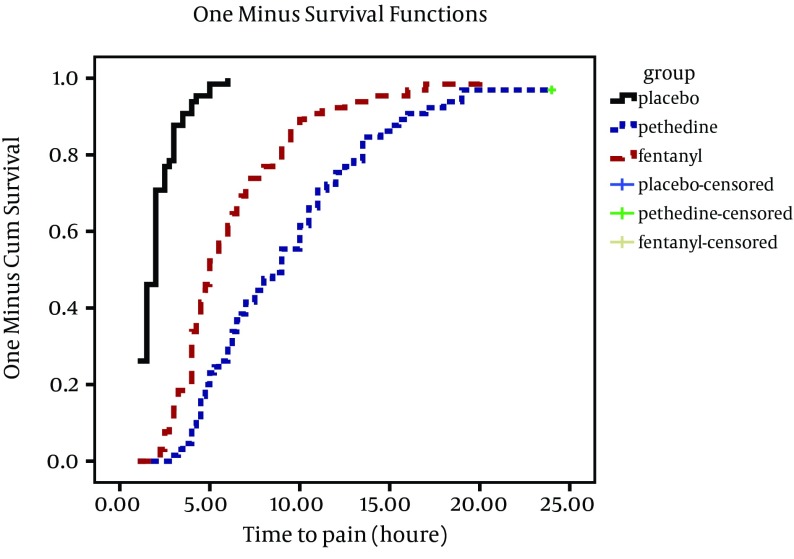
Comparing the Duration of Analgesia in the Three Groups

The mean duration of postoperative analgesia (hours) in meperidine, fentanyl, and placebo groups were 9.46 ± 0.6, 6.27 ± 0.45, 2.06 ± 0.13, respectively, (Table 2) and there were statistically significant differences among the three groups (P < 0.0001) by nonparametric Kruskal-Wallis test. Mean duration of postoperative analgesia was remarkably longer in the meperidine group. Considering the variable entity of the duration of analgesia assessed in our study, Kaplan Mayer method and log rank test were used to compare survival function of time to event (pain more than 4 by the visual analogue scale) in the three groups, and the results are shown in (Figure 1). The onset time of sensory block to T6 and complete motor block in meperidine group were less than the other two groups. Level of sensory block in meperidine group was higher than the others. Duration of sensory recovery to T10 level in meperidine group was more than the two other groups. Complete motor recovery time had no difference between fentanyl and meperidine groups, but in both groups was more than the placebo groups (P < 0.0001). All of sensory and motor block analyses were performed by Kolmogorov-Smirnov and non-parametric Kruskal-Wallis tests, and there were statistically significant differences among at least one group to the others. (Table 3) 

According to Chi Square test statistically significant differences were not observed in neonatal Apgar scores at 1 minute after birth (P = 0.45). Apgar scores at 5 minutes after birth were 8 or higher in all the three groups (Table 4)

**Table 2.** Mean Durations of Postoperative Analgesia (Hours) in the Three Groups

Group	No.	Mean Duration of Analgesia, hr	Standard deviation (SD)	Value of Test	P value
**Placebo**	65	2.06	0.13	120.5	0.0001
**Meperidine**	65	9.46	0.6	120.5	0.0001
**Fentanyl**	65	6.27	0.45	120.5	0.0001

**Table 3.** Mean Duration of Sensory and Motor Block (Min) and Sensory Level (Thoracic) in Three Groups

Variables	Data	P value
**Onset time of Sensory Block to T6, min, Mean ± SD**		< 0.0001
Placebo	5.13 ± 0.29	
Meperidine	4.5 ± 0.51	
Fentanyl	5.05 ± 0.38	
**Onset time of Complete Motor Block, min**		< 0.0001
Placebo	6.86 ± 0.7	
Meperidine	6.24 ± 0.66	
Fentanyl	6.67 ± 0.64	
**Level of Sensory Block, Thoracic**		< 0.0001
Placebo	T5 (T5-T6) 53.8	
Meperidine	T4 (T4-T5) 53.8	
Fentanyl	T5 (T5-T6) 56.9	
**Sensory Recovery Time to T10, min, Mean **±** SD**		< 0.0001
Placebo	119.8 ± 3.49	
Meperidine	158.44 ± 4.24	
Fentanyl	146.13 ± 4.54	
**Complete Motor Recovery, min, Mean **±** SD**		< 0.0001
Placebo	109.67 ± 16.2	
Meperidine	118.52 ± 4.63	
Fentanyl	115.44 ± 3.04	

**Table 4.** Apgar Scores at 1 and 5 Minutes After Birth in the Three Groups

Time	Apgar Score	Placebo, No. (%)	Meperidine, No. (%)	Fentanyl, No. (%)	Total, No. (%)	P value
**1 minute**	6 – 7	3 (4.6)	6 (9.2)	3 (4.6)	12 (6.2)	0.45
	**8 – 10**	62 (95.4)	59 (90.8)	62 (95.4)	183 (93.8)	
**5 minutes**	6 – 7	0	0	0	0	-
	**8 – 10**	65 (100)	65 (100)	65 (100)	195 (100)	
**Total**	-	65 (100)	65 (100)	65 (100)	195 (100)	-

**Table 5.** Comparing the Prevalence of Complications Between the Two Intervention Groups and Placebo (Placebo Is the Reference Group)

Groups	Placebo (N = 65), No (%)	Meperidine, (N = 65)		Fentanyl, (N = 65)		P value
		No. (%)	ARR ^a^ % (CI 95%)	No. (%)	ARR % (CI 95%)	
**Hypotension**	35 (53.8)	41 (63.1)	-9.3 (-26, 7)	35 (53.8)	-9.3 (-26, 7)	0.471
**Nausea**	22 (33.8)	18 (27.7)	6.1 (-9, 21)	20 (30.8)	3.3 (-13, 19)	0.749
**Vomiting**	9 (13.8)	6 (9.2)	4 (-6, 15)	11 (16.9)	-3.1 (-15, 9)	0.43
**Sedation**	0 (0)	2 (3.1)	-2 (-7, 1)	6 (9.2)	-9 (-16, -2)	0.026
**Pruritus **	2 (3.1)	0 (0)	-2 (-10, 4)	4 (6.2)	-9.3 (-26, 7)	0.127
**Respiratory Depression**	0 (0)	0 (0)	-9.3 (-26, 7)	1 (1.5)	-1.5 (-4.5, 1.5)	0.366

^a^ Abbreviations: ARR, absolute risk reduction.

There was no statistically significant difference in the incidence of hypotension, nausea, vomiting, pruritus, and respiratory depression among the three groups (Table 5). Absolute Risk Reduction (ARR) of complications in meperidine and fentanyl groups compared with the placebo group was also shown in Table 5. 

Statistically significant difference was found in the incidence of sedation among the three groups (P = 0.026) as the highest incidence of sedation was found in the fentanyl group, and there was no case of sedation in the placebo group.

## 5. Discussion

Adding opioids to local anesthetics has been used for over 30 years to enhance analgesia and reduce the dose of local anesthetics and therefore, their adverse effects; however, finding the appropriate opioid with the most efficacy and the least adverse effects is still controversial (10, 11). In this study, postoperative analgesia duration and adverse effects of meperidine and fentanyl were compared with placebo, and it was found that the duration of analgesia in the meperidine group was more than the two other groups and the placebo group had the shortest duration of analgesia. The incidence of side effects except for sedation did not show any significant difference among the three groups. Sedation was remarkably more common in the fentanyl group. In this study Quincke needle was used because Etezadi et al. found that, needle type had not significant impact on post spinal complications such as transient neurologic symptoms (12). 

Shrestha and colleagues (6) compared meperidine 1mg/kg with 0.5% hyperbaric bupivacaine 2.2 mL administered intrathecally in 60 patients undergoing cesarean section with spinal anesthesia. The duration of analgesia was 8.30 hours in the meperidine group and 2.36 hours in the bupivacaine group. Changes in blood pressure and heart rate and Apgar scores of the newborns at 1 and 5 minutes did not show any statistically significant difference. In the meperidine group 20% of the patients had pruritus and 10% had nausea and vomiting. There was no case of respiratory depression. The dose of used intrathecal meperidine in our study was 30% of that in Shrestha and colleagues, combined with lidocaine and epinephrine, which caused longer duration of analgesia with no case of pruritus. Hemodynamic changes and Apgar scores were similar in the both studies. Probably, reduced dose of meperidine and using epinephrine were responsible for these differences.

In the study conducted by Weigl et al. (10), duration of analgesia and adverse effects of intrathecal fentanyl were compared with intrathecal morphine added to bupivacaine for spinal anesthesia in elective cesarean section. The patients in the morphine group had longer duration of analgesia and less need for analgesic medications. Mean duration of analgesia in the fentanyl group was 3-4 hours which was shorter than that of our study (6 hours). As fentanyl dosages were the same in the both studies, it is possible that added epinephrine to the intrathecal drugs caused slower absorption and longer duration of analgesia in the fentanyl group in our study. The incidence of pruritus was 35.7% in the morphine group and 10.3% in the fentanyl group; while pruritus was far less common in our patients; 0% in the meperidine group and 6.2% in the fentanyl group. Using meperidine instead of morphine and adding epinephrine to the drug combination leading to slower absorption of the drugs probably explain this difference. Weigl et al. did not report any case of sedation, although it is not clear that which sedation scale was used (10). In our study, the highest incidence of sedation (9.2%) was found in the fentanyl group.

The incidence of respiratory depression was similar between Weigl et al. study and our study with only one case in both fentanyl groups.

Harsoor et al. (11) compared low dose bupivacaine (1.6 ml of 0.5% solution) with placebo or fentanyl (12.5 µg) for spinal anesthesia in patients undergoing elective cesarean section. The time interval between the injection of the intrathecal drug and the first request for analgesic medication by the patient was considered as the duration of analgesia. This duration was 103 minutes in the bupivacaine and placebo group, and 184 minutes in the bupivacaine and fentanyl group. No significant differences were observed with respect to maternal complications including hemodynamic changes, pruritus, sedation and respiratory depression, or neonatal Apgar scores at 1 and 5 minutes after birth. The duration of analgesia in the fentanyl group was longer in our study, about 6 hours, having in mind that our starting point of the duration of analgesia was the ending time of the operation. In our study, the highest incidence of sedation (9.2%) was found in the fentanyl group; probably as a result of the higher dose of fentanyl and adding epinephrine to the combination.

Shahriari et al. (13) compared the effect of adding low dose fentanyl (15 µg) to lidocaine with placebo in spinal anesthesia for elective cesarean section, and showed better quality of anesthesia during the operation and longer duration of postoperative analgesia in the fentanyl group. There were no significant differences with respect to maternal complications and neonatal Apgar scores. The duration of analgesia in the fentanyl group was longer in our study probably as a result of the higher dose of fentanyl and adding epinephrine to the combination.

We did not find any significant differences in the incidence of hypotension and pruritus among patients in these three groups which is consistent with the findings of Connelly et al. (14) who added fentanyl to the combination of morphine-lidocaine-epinephrine for spinal anesthesia. 

In some studies including Anaraki et al. (15) and Han et al. (16) intrathecal meperidine and fentanyl were used as prophylaxis against shivering. In the study of Anaraki, shivering was better controlled by increasing the dose of meperidine; however, the incidence of pruritus, nausea, and vomiting increased. In our study the incidence of nausea in the meperidine group was similar to the study of Anaraki, but there was no case of pruritus. The differences between these studies were also to some extent due to different types of local anesthetics.

Yu et al. (17) added meperidine to intrathecal bupivacaine for spinal anesthesia in elective cesarean section and studied the duration of analgesia and the adverse effects. The duration of analgesia was shorter than our study which would be partly due to its lower dose of meperidine; 10 mg compared to 25 mg administered in our study. In that study, the incidence of nausea and vomiting during the operation was investigated, which was higher in the meperidine group than the control group. In our study there was no statistically significant difference in the incidence of postoperative nausea and vomiting among the three groups. It is possible that nausea and vomiting during the operation is partly due to surgical manipulation and peritoneal stimulation, and eliminated When the operation was finished.

In the study by Chaudhari et al. (18) performed on patients candidates for perianal surgery under spinal anesthesia with meperidine or lidocaine, the mean duration of analgesia in the meperidine group was 15.3 hours. The incidence of hypotension in the lidocaine group was higher than the meperidine group. Generally, the incidences of adverse effects including nausea, vomiting and pruritus in the meperidine group were higher in that study. In our study the mean duration of analgesia in the meperidine group was shorter (9.46 hours) which may be due to the higher dose of meperidine (1 mg/kg) and /or the type of operations in the Chaudhari study (18). We did not find any significant difference in the incidence of nausea and vomiting and hypotension in our study between lidocaine and meperidine groups, which may be due to lower dose of meperidine in our study. There was no case of pruritus in the meperidine group in our study.

Golfam et al. (7) compared different doses of lidocaine (50-60-75 mg) combined with a fixed dose of sufentanil (2.5 µg) and epinephrine (100 µg) to find the minimum appropriate dose of hyperbaric lidocaine 5% with less complications. They concluded that nausea, vomiting and dyspnea were degraded with lower doses of lidocaine, especially 50 µg. We used lidocaine 70 mg with adjuvant. Rate of nausea and vomiting in our study was similar to group 2 (60 mg) in Golfam study. However, the rate of hypotension in Golfam study in all patients was higher than our study. It may be due to different doses of lidocaine, as well as different adjuvants in these two studies.

Characteristics of sensory and motor block in our study in saline and fentanyl groups were similar to Chung et al. (8) study. But we can see different results between our study and Bakhshaei et al. (19) study, which might be due to the different opioid (sufentanil) used in their study without epinephrine. 

According to our study, adding neuraxial meperidine or fentanyl to lidocaine and epinephrine remarkably increased the duration of postoperative analgesia after cesarean section. Finally, meperidine is recommended as an additive considering longer duration of analgesia and less adverse effects.
